# Crystal structure of *trans*-bis­(7-benzyl-1,3-dimethyl-3,7-di­hydro-1*H*-purine-2,6-dione)dichloridopalladium(II) hemihydrate

**DOI:** 10.1107/S205698902501117X

**Published:** 2026-01-01

**Authors:** Katsuya Kaikake, Sotaro Kusumoto, Ren-Hua Jin

**Affiliations:** a Department of Applied Chemistry, Faculty of Chemistry and Biochemistry, Kanagawa University, Yokohama 221-8686, Japan; Universidad de la República, Uruguay

**Keywords:** crystal structure, palladium, benzyl­theophylline, xanthine derivative

## Abstract

An air-stable palladium bis­(benzyl­theophylline) complex, [PdCl_2_(C_14_H_14_N_4_O_2_)_2_]·0.5H_2_O, was synthesized from 7-benzyl­theophylline and palladium dichloride. Single-crystal X-ray diffraction analysis revealed that the complex adopts a centrosymmetric structure in which two theophylline ligands coordinate to the square-planar Pd^II^ center through N atoms on the imidazole ring. The crystal structure contains water mol­ecules located at partially occupied sites (occupancy = 1/4). A notable structural feature is that the fused purine ring system of each ligand is oriented nearly perpendicular to the square-planar Pd^II^ coordination plane. In addition, the pendant phenyl ring is almost perpendicular to the fused purine ring plane, as indicated by the large torsion angle around the C5—N4—C8—C9 linkage [84.1 (2)°]. These geometric characteristics highlight the steric influence exerted by the benzyl­theophylline ligand on the metal coordination environment.

## Abstract 2

The crystal structure of a new complex between the theophylline ligand and palladium has been elucidated.

## 1. Chemical context

**Figure 1:**
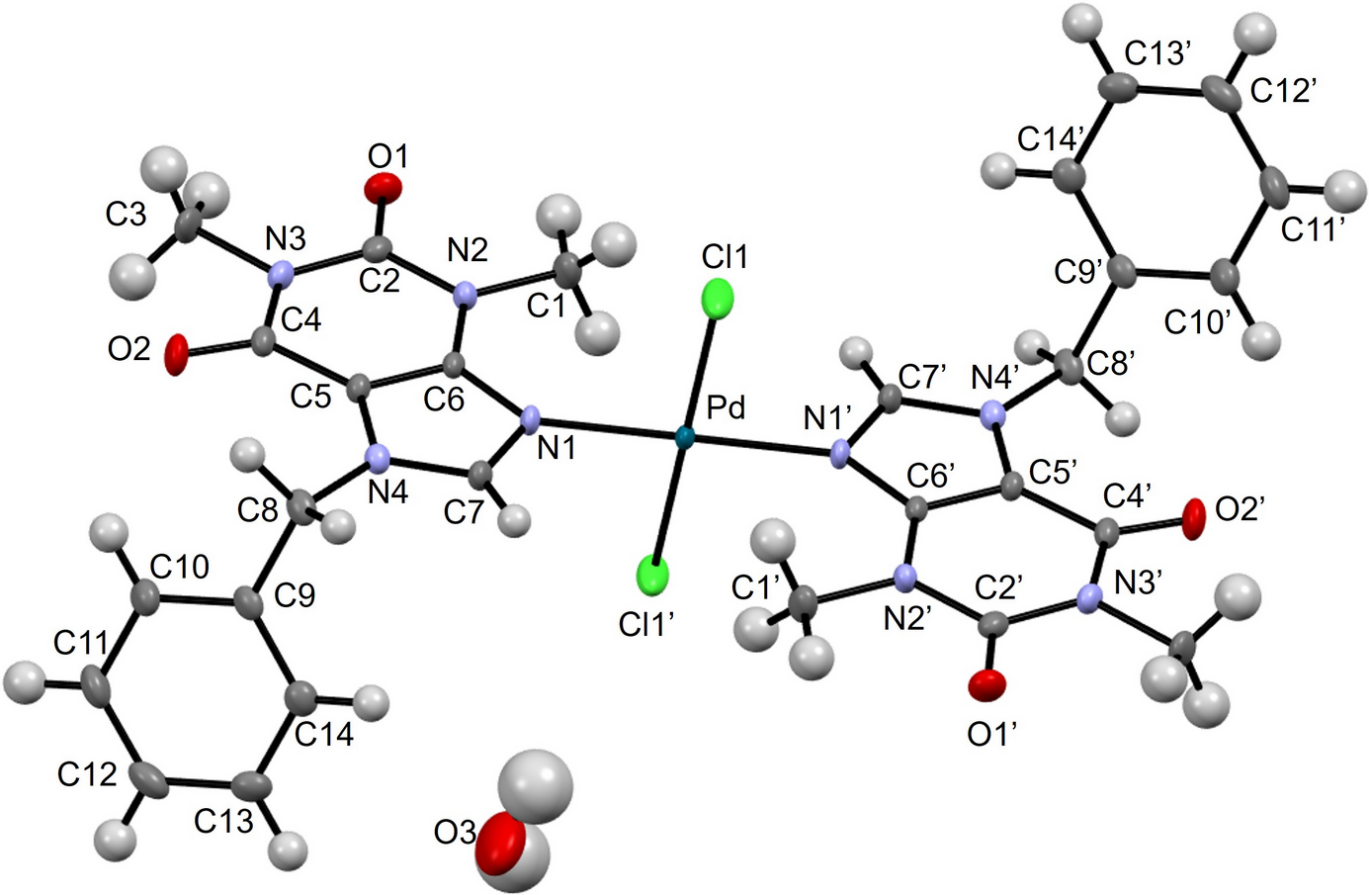
The mol­ecular structure of *trans*-bis­(7-benzyl-1,3-dimethyl-3,7-di­hydro-1*H*-purine-2,6-dione)dichloridopalladium(II) hemihydrate. Primed atoms are generated from the non-primed atoms by an inversion center [symmetry code: (′) −*x* + 1, −*y* + 1, −*z* + 1). Displacement ellipsoids are drawn at the 50% probability level.

Theophylline is a natural xanthine derivative found in cacao beans like caffeine, with a structure comprising the purine skeleton of fused pyrimidine and imidazole rings (Franco *et al.*, 2013 ▸). The structure of theophylline allows it to form N-heterocyclic carbene (NHC) complexes *via* its imidazole ring with divalent metals such as Ag^I^ and Ir^I^ (Mohamed *et al.*, 2015 ▸; Eslava-Gonzalez *et al.*, 2020 ▸). In addition, the inter­actions between the imidazole ring of theophylline and metals form nitro­gen-coordinated complexes (Gacki *et al.*, 2019 ▸, 2020 ▸; Jin *et al.*, 2019 ▸). In this sense, theophylline is a suitable ligand for metal complexation. Practically, theophylline–metal complexes have demonstrated promising potential as anti­bacterial and anti­cancer agents, thereby attracting significant attention in the pharmaceutical field (Ismail *et al.*, 2020 ▸; Gordon *et al.*, 2022 ▸). Furthermore, theophylline has been recognized as a useful ligand for palladium-catalyzed coupling reactions such as the Suzuki–Miyaura, Mizoroki–Heck, and Sonogashira reactions (Rahman *et al.*, 2022 ▸; Mazars *et al.*, 2023*a* ▸,*b* ▸; Mazars *et al.*, 2023). For examples, not only NHC complexes of theophylline derivatives (Tyagi, *et al.*, 2020 ▸; Charbonneau *et al.*, 2014 ▸; Feng *et al.*, 2014 ▸; Gazvoda *et al.*, 2016 ▸), but also N-chelated palladium catalysts derived from theophylline (Kaikake *et al.*, 2018 ▸, 2021 ▸, 2023 ▸) can effectively promote C—C coupling reactions. However, in our previous studies, the heterogeneous theophylline–palladium catalysts did not yield single crystals suitable for structural analysis, leaving their coordination structures unresolved. In contrast, the benzyl-substituted theophylline derivative used in the present study exhibits good solubility and enables the growth of high-quality crystals. Therefore, to further understand the coordination preferences of benzyl-substituted theophylline ligands toward Pd^II^ and to expand the structural information available for this class of purine-based metal complexes, we synthesized and structurally characterized the title compound (Fig. 1 ▸).
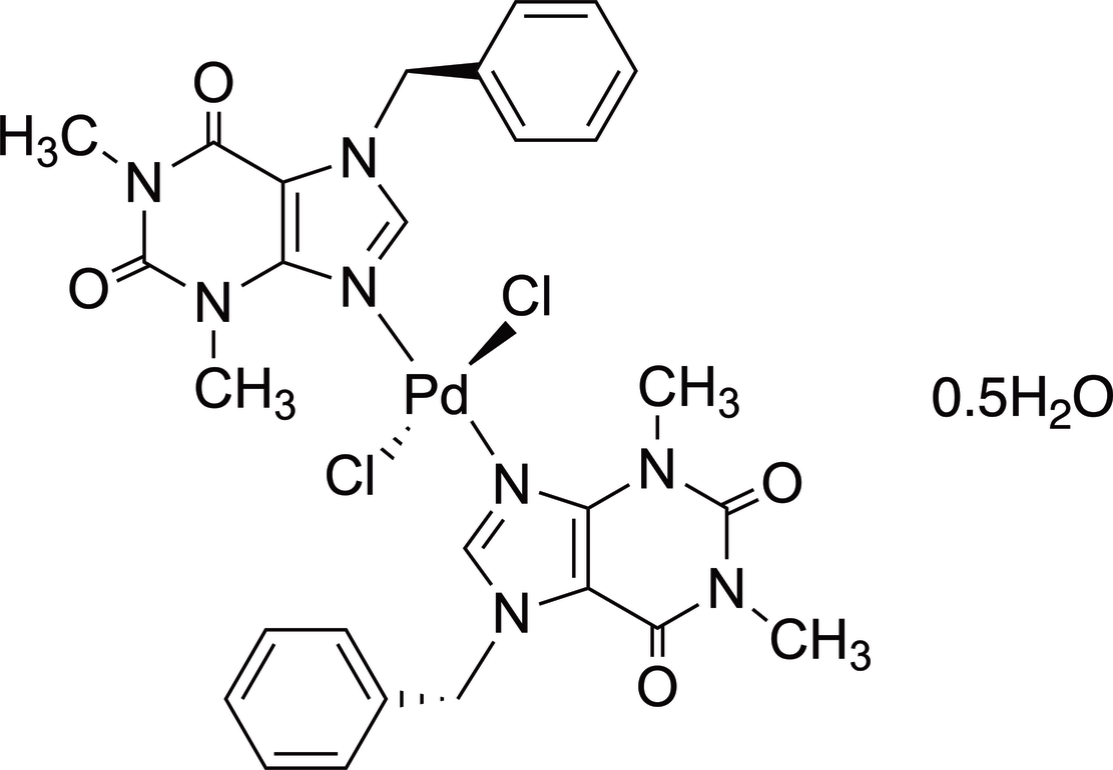


## 2. Structural commentary

Slow vapor diffusion of methanol into a chloro­form solution of the PdBzT complex produced yellow prismatic crystals suitable for X-ray analysis. The asymmetric unit contains one half of a centrosymmetric Pd^II^ complex mol­ecule, where the metal center sits on a crystallographic center of inversion, plus one site for a water mol­ecule, which, unusually, is only 25% occupied, as confirmed by refinement of the site occupation factor of the water oxygen atom. The X-ray structure revealed the expected discrete complex, crystallized with a half water solvate mol­ecule in the monoclinic space group *P*21/*c*. The Pd^II^ center adopts a square-planar coordination geometry defined by two nitro­gen atoms (N1 and N1′) from two theophylline-derived imidazole rings and two trans-arranged chloride ligands. The Pd—N and Pd—Cl bond lengths are 2.0158 (16) and 2.2880 (5) Å, respectively. The Cl1—Pd—N1 and Cl1—Pd—N1′ bond angles are 89.50 (5) and 90.50 (5)°, respectively. Each ligand features a nearly perpendicular orientation between the fused purine ring system and the square-planar coordination plane. In this complex, the phenyl ring is oriented almost perpendicular to the relevant mol­ecular planes. The plane of the fused rings system of the ligand lies nearly perpendicular to the square-planar coordination plane that includes the chloride ligands. In addition, the phenyl ring is itself almost perpendicular to the fused ring plane. More specifically, the C8—C9 bond is twisted out of the fused-ring plane by approximately 90° (observed: 84°). The torsion angle around the N4—C8 bond is 84.1 (2)°. The coordination environment of the Pd^II^ center is consistent with that commonly observed for other square-planar complexes containing N-donor ligands, and is structurally comparable to related theophylline metal complexes reported in the Cambridge Structural Database (CSD Version 6.00, last update in August of 2025; Groom *et al.*, 2016 ▸), including the octa­hedral tetra­aqua Mg^II^, Ca^II^, Mn^II^, Co^II^, Ni^II^, and Cd^II^ complexes, as well as the Cu^II^ chloride complex, although their auxiliary ligands differ (all except the Cu complex possess tetra­aqua coordination; Shi & Lou, 2015 ▸; Hao *et al.*, 2018 ▸; El Hamdani *et al.*, 2017 ▸; Gacki *et al.*, 2019 ▸; Buncel *et al.*, 1985 ▸; Biagini Cingi *et al.*, 1983 ▸).

## 3. Supra­molecular features

**Figure 2:**
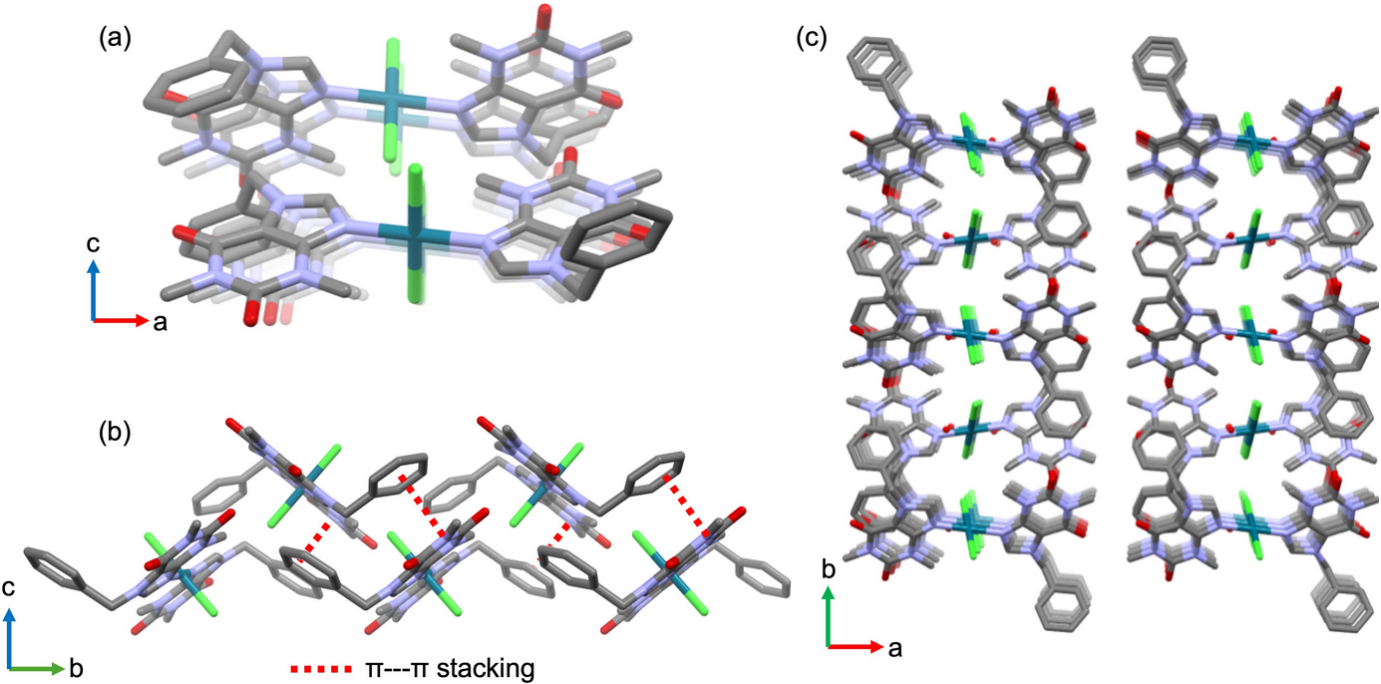
(*a*) Inter­molecular π–π stacking structure viewed along the *b* axis ([010] direction), and (*b*) the corresponding view along the *a* axis. (*c*) Packing structure viewed along the *c* axis.

The phenyl, imidazole, and di­methyl­uracil groups in the ligand are positioned obliquely on the *ab* plane and all aligned parallel to each other along the *c*-axis. No significant π–π inter­actions are observed between identical moieties within the crystal structure. However, π–π inter­actions are present between the phenyl and di­methyl­uracil groups [C2⋯C13, 3.205 (3) Å; centroid–centroid distance, 3.6452 (12) Å]. These inter­molecular π–π stacking inter­actions propagate in a zigzag manner along the [010] direction (Fig. 2 ▸). The mol­ecules also exhibit various supra­molecular inter­actions [O1⋯C12, 3.313 (3) Å; O1⋯C13, 3.394 (3) Å; C8⋯C13, 3.350 (3) Å; C7⋯Cl1, 3.514 (2) Å] with neighboring mol­ecules. Water mol­ecules inter­act with the chloro ligand [O3′⋯Cl1, 3.133 (1), O3⋯Cl1, 3.163 (1) Å] and the imidazole moiety [O3⋯N1, 3.566 (1), O3⋯C7=3.154 (1) Å].

## 4. Database survey

A search of the Cambridge Structural Database (CSD Version 6.00, last update in August of 2025; Groom *et al.*, 2016 ▸) was conducted using theophylline as a keyword, focusing on metal complexes. The structures most closely related to the present complex are tetra­aqua­bis­(1,3-dimethyl-2,6-dioxo-1,2,3,6-tetrahydro-7*H*-purin-7-yl)cobalt(II) (El Hamdani *et al.*, 2017 ▸), tetra­aqua­bis­(1,3-dimethyl-2,6-dioxo-3,7-di­hydro-1*H*-purin-9-ido)magnesium (Shi & Lou, 2015 ▸), and an anhydrous theophylline–copper(I) chloride complex (Biagini Cingi *et al.*, 1983 ▸). Notably, metal complexes in which theophylline is coordinated through the N9 atom of the imidazole ring typically adopt a *trans* square-planar coordination environment, which further supports the structural assignment of the present Pd^II^ complex. The survey also revealed that the crystal structure of bis(7-benzyl-1,3-dimethyl-3,7-di­hydro-1*H*-purine-2,6-dione)palladium(II) dichloride has not been reported previously.

## 5. Synthesis and crystallization

To a 500 mL pear-shaped flask equipped with a condenser, methanol (500 mL), K_2_CO_3_ (9.67 g, 70.0 mmol), and theophylline (11.62 g, 64.5 mmol) were added. Benzyl chloride (7.59 g, 59.9 mmol) was then introduced, and the mixture was refluxed at 353 K for 48 h. Afterwards, the solvent was removed under reduced pressure, and the residue was extracted with chloro­form and water. The chloro­form layer was concentrated, and the resulting crude product 7-benzyl­theophylline (BzT) was recrystallized from an *n*-hexa­ne/ethyl acetate mixture to yield a white crystalline powder (8.32 g, 51.3% isolated yield). Complexation of BzT and palladium(II) chloride was carried out in a water–ethanol system. An ethanol solution of BzT (5.0 mmol dm^−3^) was prepared by dissolving 337.6 mg of BzT in 250 mL of analytical-grade ethanol. An aqueous solution of PdCl_2_ (5.0 mmol dm^−3^) was prepared by dissolving palladium(II) chloride in 1.0 *M* hydro­chloric acid. Equal volumes (250 mL each) of the two solutions were mixed and allowed to stand for ten days. The yellow precipitates (PdBzT) that formed spontaneously was collected by filtration, washed alternatively with water and ethanol, and dried under ambient conditions to give PdBzT in 83.4% yield (0.3744 g). Single crystals suitable for X-ray analysis were obtained by vapor-diffusion crystallization, in which a chloro­form solution of the PdBzT complex placed in a small sample vial was allowed to slowly equilibrate with methanol vapor in a larger sealed sample vial at room temperature.

## 6. Refinement

Crystal data, data collection and structure refinement details are summarized in Table 1 ▸. All H atoms, except those of the partial occupancy water mol­ecule were located in a difference-Fourier map. Then the methyl H atoms were constrained to an ideal geometry (C—H = 0.98 Å), with *U*_iso_(H) = 1.5*U*_eq_(C), and were allowed to rotate freely about the parent N—C bonds. All other H atoms were placed in geometrically idealized positions and constrained to ride on their parent atoms, with C—H distances of 0.95 (aromatic, alkene) or 0.99 Å (methyl­ene), and *U*_iso_(H) = 1.2*U*_eq_(C). An ideal geometry for the H atoms of the water mol­ecule was generated (O—H = 0.87 Å) and then the orientation and position of the water mol­ecule were refined as a rigid group with *U*_iso_(H) = 1.5*U*_eq_(O).

## Supplementary Material

Crystal structure: contains datablock(s) I. DOI: 10.1107/S205698902501117X/oo2016sup1.cif

Structure factors: contains datablock(s) I. DOI: 10.1107/S205698902501117X/oo2016Isup2.hkl

Supporting information file. DOI: 10.1107/S205698902501117X/oo2016Isup3.mol

CCDC reference: 2515121

Additional supporting information:  crystallographic information; 3D view; checkCIF report

## Figures and Tables

**Table 1:** Experimental details

Crystal data	
Chemical formula	[PdCl_2_(C_14_H_14_N_4_O_2_)_2_]·0.5H_2_O
*M*_r_	726.89
Crystal system, space group	Monoclinic, *P*2_1_/*c*
Temperature (K)	120
*a*, *b*, *c* (Å)	16.1659 (5), 11.0124 (4), 8.2789 (3)
β (°)	92.491 (3)
*V* (Å^3^)	1472.46 (9)
*Z*	2
Radiation type	Mo *K*α
μ (mm^−1^)	0.86
Crystal size (mm)	0.67 × 0.51 × 0.16

Data collection	
Diffractometer	ROD, SynergyCustom system, HyPix
Absorption correction	Multi-scan (*CrysAlis PRO*; Rigaku OD, 2025 ▸)
*T*_min_, *T*_max_	0.437, 1.000
No. of measured, independent and observed [*I* > 2σ(*I*)] reflections	13402, 3679, 3184
*R*_int_	0.036
(sin θ/λ)_max_ (Å^−1^)	0.720

Refinement	
*R*[*F*^2^ > 2σ(*F*^2^)], *wR*(*F*^2^), *S*	0.030, 0.077, 1.08
No. of reflections	3679
No. of parameters	210
H-atom treatment	H-atom parameters constrained
Δρ_max_, Δρ_min_ (e Å^−3^)	0.58, −1.48

Computer programs: *CrysAlis PRO* (Rigaku OD, 2025 ▸), *SHELXT2018/2* (Sheldrick, 2015*a* ▸), *SHELXL2025/1* (Sheldrick, 2015*b* ▸) and *OLEX2* (Dolomanov *et al.*, 2009 ▸).

## supplementary crystallographic information

### trans-Bis(7-benzyl-1,3-dimethyl-3,7-dihydro-1H-purine-2,6-dione)dichloridopalladium(II) hemihydrate Crystal data

**Table:**

[PdCl_2_(C_14_H_14_N_4_O_2_)_2_]·0.5H_2_O	*F*(000) = 738
*M**_r_* = 726.89	*D*_x_ = 1.639 Mg m^−^^3^
Monoclinic, *P*2_1_/*c*	Mo *K*α radiation, λ = 0.71073 Å
*a* = 16.1659 (5) Å	Cell parameters from 8350 reflections
*b* = 11.0124 (4) Å	θ = 2.5–31.1°
*c* = 8.2789 (3) Å	µ = 0.86 mm^−^^1^
β = 92.491 (3)°	*T* = 120 K
*V* = 1472.46 (9) Å^3^	Plate, clear light yellow
*Z* = 2	0.67 × 0.51 × 0.16 mm

### trans-Bis(7-benzyl-1,3-dimethyl-3,7-dihydro-1H-purine-2,6-dione)dichloridopalladium(II) hemihydrate Data collection

**Table:**

ROD, SynergyCustom system, HyPix diffractometer	3184 reflections with *I* > 2σ(*I*)
Detector resolution: 10.0000 pixels mm^-1^	*R*_int_ = 0.036
ω scans	θ_max_ = 30.8°, θ_min_ = 2.2°
Absorption correction: multi-scan (CrysAlisPro; Rigaku OD, 2025)	*h* = −20→22
*T*_min_ = 0.437, *T*_max_ = 1.000	*k* = −15→12
13402 measured reflections	*l* = −10→10
3679 independent reflections	

### trans-Bis(7-benzyl-1,3-dimethyl-3,7-dihydro-1H-purine-2,6-dione)dichloridopalladium(II) hemihydrate Refinement

**Table:**

Refinement on *F*^2^	0 restraints
Least-squares matrix: full	Hydrogen site location: mixed
*R*[*F*^2^ > 2σ(*F*^2^)] = 0.030	H-atom parameters constrained
*wR*(*F*^2^) = 0.077	*w* = 1/[σ^2^(*F*_o_^2^) + (0.034*P*)^2^ + 1.0985*P*] where *P* = (*F*_o_^2^ + 2*F*_c_^2^)/3
*S* = 1.08	(Δ/σ)_max_ < 0.001
3679 reflections	Δρ_max_ = 0.57 e Å^−^^3^
210 parameters	Δρ_min_ = −1.48 e Å^−^^3^

### trans-Bis(7-benzyl-1,3-dimethyl-3,7-dihydro-1H-purine-2,6-dione)dichloridopalladium(II) hemihydrate Special details

**Table:**

Geometry. All esds (except the esd in the dihedral angle between two l.s. planes) are estimated using the full covariance matrix. The cell esds are taken into account individually in the estimation of esds in distances, angles and torsion angles; correlations between esds in cell parameters are only used when they are defined by crystal symmetry. An approximate (isotropic) treatment of cell esds is used for estimating esds involving l.s. planes.

### trans-Bis(7-benzyl-1,3-dimethyl-3,7-dihydro-1H-purine-2,6-dione)dichloridopalladium(II) hemihydrate Fractional atomic coordinates and isotropic or equivalent isotropic displacement parameters (Å^2^)

**Table:**

	*x*	*y*	*z*	*U*_iso_*/*U*_eq_	Occ. (<1)
Pd1	0.500000	0.500000	0.500000	0.01424 (7)	
Cl1	0.52069 (4)	0.36420 (6)	0.29652 (8)	0.03618 (16)	
O2	0.09973 (9)	0.53276 (15)	0.3149 (2)	0.0244 (3)	
O1	0.20421 (10)	0.24243 (13)	0.6623 (2)	0.0247 (3)	
N1	0.38153 (10)	0.52341 (15)	0.4199 (2)	0.0156 (3)	
N2	0.29723 (10)	0.36958 (14)	0.5516 (2)	0.0163 (3)	
N4	0.27897 (10)	0.61942 (14)	0.28786 (19)	0.0149 (3)	
N3	0.15297 (10)	0.39104 (15)	0.4934 (2)	0.0177 (3)	
C7	0.36052 (12)	0.61387 (17)	0.3181 (2)	0.0169 (4)	
H7	0.399162	0.667715	0.272902	0.020*	
C6	0.30856 (11)	0.46807 (17)	0.4547 (2)	0.0143 (4)	
C9	0.20311 (13)	0.81710 (18)	0.2648 (2)	0.0167 (4)	
C2	0.21744 (13)	0.32854 (17)	0.5748 (2)	0.0180 (4)	
C5	0.24408 (12)	0.52649 (17)	0.3751 (2)	0.0144 (4)	
C1	0.36631 (13)	0.30639 (19)	0.6358 (3)	0.0240 (4)	
H1A	0.406034	0.279681	0.557080	0.036*	
H1B	0.345246	0.235608	0.692869	0.036*	
H1C	0.393811	0.361640	0.713879	0.036*	
C10	0.11867 (13)	0.8273 (2)	0.2860 (3)	0.0225 (4)	
H10	0.082185	0.764931	0.248108	0.027*	
C3	0.06885 (13)	0.3462 (2)	0.5180 (3)	0.0257 (5)	
H3A	0.048881	0.300923	0.422158	0.039*	
H3B	0.031962	0.415139	0.535590	0.039*	
H3C	0.069598	0.292707	0.612631	0.039*	
C4	0.15987 (12)	0.48838 (17)	0.3861 (3)	0.0167 (4)	
C14	0.25624 (13)	0.90993 (18)	0.3189 (2)	0.0199 (4)	
H14	0.313889	0.904545	0.302186	0.024*	
C8	0.23679 (13)	0.70838 (18)	0.1788 (2)	0.0180 (4)	
H8A	0.190586	0.667191	0.118481	0.022*	
H8B	0.276373	0.736720	0.098959	0.022*	
C11	0.08731 (14)	0.9291 (2)	0.3629 (3)	0.0274 (5)	
H11	0.029463	0.936317	0.376421	0.033*	
C13	0.22463 (16)	1.01019 (18)	0.3974 (3)	0.0231 (4)	
H13	0.260869	1.072603	0.436224	0.028*	
C12	0.14025 (16)	1.0193 (2)	0.4192 (3)	0.0269 (5)	
H12	0.118867	1.087895	0.473029	0.032*	
O3	0.4221 (7)	1.0115 (9)	0.5025 (14)	0.066 (3)	0.25
H3D	0.443268	0.943429	0.470289	0.099*	0.25
H3E	0.424101	1.006224	0.607442	0.099*	0.25

### trans-Bis(7-benzyl-1,3-dimethyl-3,7-dihydro-1H-purine-2,6-dione)dichloridopalladium(II) hemihydrate Atomic displacement parameters (Å^2^)

**Table:**

	*U*^11^	*U*^22^	*U*^33^	*U*^12^	*U*^13^	*U*^23^
Pd1	0.00659 (11)	0.01241 (11)	0.02364 (13)	0.00008 (6)	−0.00017 (8)	−0.00300 (7)
Cl1	0.0191 (3)	0.0424 (3)	0.0466 (4)	0.0064 (2)	−0.0029 (2)	−0.0285 (3)
O2	0.0108 (7)	0.0318 (8)	0.0302 (9)	0.0021 (6)	−0.0030 (6)	0.0000 (7)
O1	0.0232 (8)	0.0183 (7)	0.0328 (9)	−0.0043 (6)	0.0041 (6)	0.0019 (6)
N1	0.0071 (7)	0.0161 (8)	0.0235 (9)	0.0004 (6)	−0.0008 (6)	−0.0006 (6)
N2	0.0105 (8)	0.0146 (8)	0.0237 (9)	0.0007 (6)	−0.0005 (6)	0.0005 (6)
N4	0.0122 (8)	0.0170 (8)	0.0155 (8)	0.0012 (6)	0.0009 (6)	−0.0011 (6)
N3	0.0107 (8)	0.0181 (8)	0.0243 (9)	−0.0019 (6)	0.0022 (6)	−0.0030 (6)
C7	0.0098 (9)	0.0162 (9)	0.0248 (10)	−0.0002 (7)	0.0014 (7)	−0.0009 (7)
C6	0.0087 (8)	0.0142 (8)	0.0200 (10)	0.0005 (7)	0.0006 (7)	−0.0037 (7)
C9	0.0188 (10)	0.0184 (9)	0.0128 (9)	0.0052 (7)	−0.0013 (7)	0.0031 (7)
C2	0.0153 (9)	0.0159 (9)	0.0231 (10)	−0.0022 (7)	0.0026 (8)	−0.0051 (7)
C5	0.0103 (9)	0.0161 (9)	0.0167 (9)	0.0000 (7)	−0.0003 (7)	−0.0010 (7)
C1	0.0168 (10)	0.0212 (10)	0.0335 (12)	0.0008 (8)	−0.0040 (9)	0.0074 (8)
C10	0.0179 (10)	0.0246 (11)	0.0244 (11)	0.0039 (8)	−0.0041 (8)	−0.0007 (8)
C3	0.0105 (9)	0.0236 (11)	0.0431 (14)	−0.0035 (8)	0.0032 (9)	0.0008 (9)
C4	0.0108 (9)	0.0190 (10)	0.0202 (10)	0.0010 (7)	0.0009 (7)	−0.0047 (7)
C14	0.0202 (10)	0.0203 (10)	0.0194 (10)	0.0007 (8)	0.0031 (8)	0.0048 (7)
C8	0.0181 (10)	0.0230 (10)	0.0129 (9)	0.0051 (8)	−0.0001 (7)	0.0004 (7)
C11	0.0193 (11)	0.0310 (12)	0.0319 (12)	0.0101 (9)	0.0009 (9)	0.0001 (9)
C13	0.0298 (12)	0.0160 (10)	0.0234 (11)	−0.0028 (8)	0.0021 (9)	0.0020 (7)
C12	0.0340 (13)	0.0211 (10)	0.0257 (12)	0.0109 (9)	0.0029 (10)	0.0004 (8)
O3	0.045 (6)	0.087 (8)	0.063 (7)	0.000 (5)	−0.013 (5)	−0.025 (5)

### trans-Bis(7-benzyl-1,3-dimethyl-3,7-dihydro-1H-purine-2,6-dione)dichloridopalladium(II) hemihydrate Geometric parameters (Å, º)

**Table:**

Pd1—Cl1^i^	2.2880 (5)	C9—C8	1.507 (3)
Pd1—Cl1	2.2881 (5)	C5—C4	1.431 (3)
Pd1—N1	2.0158 (16)	C1—H1A	0.9800
Pd1—N1^i^	2.0158 (16)	C1—H1B	0.9800
O2—C4	1.217 (3)	C1—H1C	0.9800
O1—C2	1.218 (3)	C10—H10	0.9500
N1—C7	1.339 (3)	C10—C11	1.395 (3)
N1—C6	1.369 (2)	C3—H3A	0.9800
N2—C6	1.366 (3)	C3—H3B	0.9800
N2—C2	1.388 (3)	C3—H3C	0.9800
N2—C1	1.466 (3)	C14—H14	0.9500
N4—C7	1.333 (2)	C14—C13	1.390 (3)
N4—C5	1.387 (2)	C8—H8A	0.9900
N4—C8	1.479 (2)	C8—H8B	0.9900
N3—C2	1.397 (3)	C11—H11	0.9500
N3—C3	1.469 (3)	C11—C12	1.379 (3)
N3—C4	1.400 (3)	C13—H13	0.9500
C7—H7	0.9500	C13—C12	1.387 (3)
C6—C5	1.369 (3)	C12—H12	0.9500
C9—C10	1.389 (3)	O3—H3D	0.8702
C9—C14	1.397 (3)	O3—H3E	0.8702
			
Cl1^i^—Pd1—Cl1	180.0	N2—C1—H1C	109.5
N1—Pd1—Cl1^i^	89.50 (5)	H1A—C1—H1B	109.5
N1^i^—Pd1—Cl1^i^	90.50 (5)	H1A—C1—H1C	109.5
N1^i^—Pd1—Cl1	89.50 (5)	H1B—C1—H1C	109.5
N1—Pd1—Cl1	90.50 (5)	C9—C10—H10	119.9
N1—Pd1—N1^i^	180.0	C9—C10—C11	120.1 (2)
C7—N1—Pd1	120.90 (13)	C11—C10—H10	119.9
C7—N1—C6	105.44 (16)	N3—C3—H3A	109.5
C6—N1—Pd1	133.53 (14)	N3—C3—H3B	109.5
C6—N2—C2	119.28 (16)	N3—C3—H3C	109.5
C6—N2—C1	122.50 (16)	H3A—C3—H3B	109.5
C2—N2—C1	118.20 (17)	H3A—C3—H3C	109.5
C7—N4—C5	106.84 (16)	H3B—C3—H3C	109.5
C7—N4—C8	124.85 (17)	O2—C4—N3	122.07 (19)
C5—N4—C8	128.30 (16)	O2—C4—C5	126.37 (19)
C2—N3—C3	116.32 (17)	N3—C4—C5	111.55 (17)
C2—N3—C4	127.21 (17)	C9—C14—H14	120.1
C4—N3—C3	116.41 (17)	C13—C14—C9	119.9 (2)
N1—C7—H7	124.0	C13—C14—H14	120.1
N4—C7—N1	111.99 (17)	N4—C8—C9	113.83 (15)
N4—C7—H7	124.0	N4—C8—H8A	108.8
N1—C6—C5	109.53 (18)	N4—C8—H8B	108.8
N2—C6—N1	127.99 (18)	C9—C8—H8A	108.8
N2—C6—C5	122.48 (17)	C9—C8—H8B	108.8
C10—C9—C14	119.62 (19)	H8A—C8—H8B	107.7
C10—C9—C8	120.11 (18)	C10—C11—H11	120.0
C14—C9—C8	120.24 (18)	C12—C11—C10	120.0 (2)
O1—C2—N2	121.62 (19)	C12—C11—H11	120.0
O1—C2—N3	121.57 (19)	C14—C13—H13	119.9
N2—C2—N3	116.81 (17)	C12—C13—C14	120.1 (2)
N4—C5—C4	131.25 (18)	C12—C13—H13	119.9
C6—C5—N4	106.21 (16)	C11—C12—C13	120.2 (2)
C6—C5—C4	122.52 (18)	C11—C12—H12	119.9
N2—C1—H1A	109.5	C13—C12—H12	119.9
N2—C1—H1B	109.5	H3D—O3—H3E	104.5
			
Pd1—N1—C7—N4	−175.74 (13)	C2—N3—C4—C5	−4.4 (3)
Pd1—N1—C6—N2	−5.5 (3)	C5—N4—C7—N1	−0.1 (2)
Pd1—N1—C6—C5	174.85 (14)	C5—N4—C8—C9	84.1 (2)
N1—C6—C5—N4	0.6 (2)	C1—N2—C6—N1	−0.1 (3)
N1—C6—C5—C4	179.19 (18)	C1—N2—C6—C5	179.50 (19)
N2—C6—C5—N4	−179.04 (17)	C1—N2—C2—O1	−0.1 (3)
N2—C6—C5—C4	−0.5 (3)	C1—N2—C2—N3	179.69 (17)
N4—C5—C4—O2	0.9 (4)	C10—C9—C14—C13	1.7 (3)
N4—C5—C4—N3	−178.47 (19)	C10—C9—C8—N4	−104.9 (2)
C7—N1—C6—N2	178.96 (19)	C10—C11—C12—C13	1.0 (4)
C7—N1—C6—C5	−0.7 (2)	C3—N3—C2—O1	−0.7 (3)
C7—N4—C5—C6	−0.3 (2)	C3—N3—C2—N2	179.56 (18)
C7—N4—C5—C4	−178.7 (2)	C3—N3—C4—O2	−1.0 (3)
C7—N4—C8—C9	−97.0 (2)	C3—N3—C4—C5	178.39 (17)
C6—N1—C7—N4	0.5 (2)	C4—N3—C2—O1	−177.84 (19)
C6—N2—C2—O1	−178.70 (18)	C4—N3—C2—N2	2.4 (3)
C6—N2—C2—N3	1.1 (3)	C14—C9—C10—C11	−0.8 (3)
C6—C5—C4—O2	−177.3 (2)	C14—C9—C8—N4	77.2 (2)
C6—C5—C4—N3	3.4 (3)	C14—C13—C12—C11	−0.1 (3)
C9—C10—C11—C12	−0.6 (3)	C8—N4—C7—N1	−179.21 (17)
C9—C14—C13—C12	−1.3 (3)	C8—N4—C5—C6	178.74 (17)
C2—N2—C6—N1	178.45 (19)	C8—N4—C5—C4	0.4 (3)
C2—N2—C6—C5	−1.9 (3)	C8—C9—C10—C11	−178.76 (19)
C2—N3—C4—O2	176.16 (19)	C8—C9—C14—C13	179.66 (18)

Symmetry code: (i) −*x*+1, −*y*+1, −*z*+1.
